# Emerging Roles for NLRC5 in Immune Diseases

**DOI:** 10.3389/fphar.2019.01352

**Published:** 2019-11-19

**Authors:** Jie-quan Wang, Ya-ru Liu, Quan Xia, Ruo-nan Chen, Jun Liang, Qing-rong Xia, Jun Li

**Affiliations:** ^1^Department of Pharmacy, Hefei Fourth People’s Hospital, Hefei, China; ^2^Department of Pharmacy, Anhui Mental Health Center, Hefei, China; ^3^Department of Pharmacy, Affiliated Psychological Hospital of Anhui Medical University, Hefei, China; ^4^School of Pharmacy, Anhui Medical University, Ministry of Education, Hefei, China; ^5^Department of Pharmacy, The First Affiliated Hospital of Anhui Medical University, Hefei, China

**Keywords:** NLRC5, NOD-like receptors, immune diseases, biological functions, signaling pathways

## Abstract

Innate immunity activates the corresponding immune response relying on multiple pattern recognition receptors (PRRs) that includes pattern recognition receptors (PRRs), like NOD-like receptors (NLRs), RIG-I-like receptors (RLRs), and C-type lectin receptors (CLRs), which could accurately recognize invasive pathogens. In particular, NLRs belong to a large protein family of pattern recognition receptors in the cytoplasm, where they are highly correlated with activation of inflammatory response system followed by rapid clearance of invasive pathogens. Among the NLRs family, NLRC5, also known as NOD4 or NOD27, accounts for a large proportion and involves in immune responses far and wide. Notably, in the above response case of inflammation, the expression of NLRC5 remarkably increased in immune cells and immune-related tissues. However, the evidence for higher expression of NLRC5 in immune disease still remains controversial. It is noted that the growing evidence further accounts for the participation of NLRC5 in the innate immune response and inflammatory diseases. Moreover, NLRC5 has also been confirmed to exert a critical role in the control of regulatory diverse signaling pathways. Together with its broad participation in the occurrence and development of immune diseases, NLRC5 can be consequently treated as a potential therapeutic target. Nevertheless, the paucity of absolute understanding of intrinsic characteristics and underlying mechanisms of NLRC5 still make it hard to develop targeting drugs. Therefore, current summary about NLRC5 information is indispensable. Herein, current knowledge of NLRC5 is summarized, and research advances in terms of NLRC5 in characteristics, biological function, and regulatory mechanisms are reviewed.

## Introduction

The innate immune system has always been a striker against pathogenic microorganisms such as bacteria, fungi, and viruses (Akira et al., 2006; Medzhitov, 2007). Innate immunity is also responsible for most inflammatory responses, and the stress of innate immunity also reflects the status of the pathogen-specific adaptive immunity (Janeway and Medzhitov, 2002; Beutler, 2004). Innate immunity activates the corresponding immune response, requiring multiple pattern recognition receptors (PRRs) that recognize the molecular structures of specific pathogens in different cellular components, such as the cytoplasmic membrane, endosomes, and cytoplasm (Janeway, 1989; Kumar et al., 2009). To date, it was demonstrated that multiple families of PRRs includes NOD-like receptors (NLRs), Toll-like receptors (TLRs), RIG-I-like receptors (RLRs) and C-type lectin receptors (CLRs) (Inohara et al., 2005; Meylan and Tschopp, 2006; Kanneganti et al., 2007; Lamkanfi and Dixit, 2009). Despite relatively large differences in their structures, expressions, and localizations, all of them can specifically recognize invading pathogens and further initiate the innate and adaptive immune response when organism suffers from intrusion of non-self’s pathogens (**Table 1**) (Yoneyama and Fujita, 2009; Dixit and Kagan, 2013; Dambuza and Brown, 2015; Kouli et al., 2019). Among PRRs family, NLRs account for a large proportion in the cytoplasm, where involves in inflammatory diseases and promotes the rapid clearance of invasive pathogens (Xu et al., 2015; Yang et al., 2016). It was reported that NLRC5 is one of the largest members of the NLRs family, which could modulate inflammatory responses and many other human diseases (Rodriguez et al., 2016; Qiu et al., 2017). It has been demonstrated that the expression of NLRC5 increased significantly in immune cells and immune-related tissues of inflammatory response case, and NLRC5 also showed contradictory roles in the modulation of immune response (Benko et al., 2010; Cui et al., 2010; Yao and Qian, 2013). Accordingly, NLRC5 is emerging as a novel therapeutic target, which is rapidly attracting more extensive attention in many other inflammatory diseases. Meanwhile, accumulating evidence suggested that NLRC5 plays vital regulatory role in the occurrence and development of immune diseases. Therefore, a thorough understanding of the biological functions and potential molecular mechanisms of NLRC5 may give us inspirations on the development of therapeutic drugs targeting NLRC5. Following recent advances, this paper aims to provide a comprehensive update on the diverse pharmacological roles of NLRC5 in the process of immune diseases, especially on liver diseases, renal diseases, rheumatoid arthritis, heart diseases, lung diseases, and spleen diseases.

**Table 1:** Implications (structure, expression, and location) of PRRs family.

PRRs	Representative protein	Structure	Expression	Location	Involvements	References
NLRs	NLRC5	Nucleotide binding domains, Leucine-rich repeat genes, Triple domain	Primary cells of myeloid and lymphoid origin, Bone marrow, human THP-1 cells, B cells, human cervical cancer cell line	Cytoplasm/nucleus	23 NLR genes in human genome, 34 NLR genes in mice genome	Harton et al., 2002; Inohara and Nuñez, 2003
TLRs	TLR5	Leucine-rich repeat domain	Membrane of Gram-negative bacteria, Membrane of endosomal lysosomal compartment	Plasma membrane/intracellular compartment	10 TLR genes in human genome, 12 TLR genes in mice genome	Kouli et al., 2019
RLRs	MDA5	Carbohydrate recognition domain	Low levels in resting cells	Cytoplasm	Unknown	Dixit and Kagan, 2013; Yoneyama and Fujita, 2009
CLRs	Dectin-1	C-type lectin-like domain	primary expressed in myeloid cells	Cytoplasm	Unknown	Dambuza and Brown, 2015

## Structure, Expression, and Localization

At present, there are 23 NLR genes in the human genome and at least 34 NLR genes in the mouse genome that are expressed widely in various cells and tissues (Harton et al., 2002; Inohara and Nunez, 2003). It is commonly known that the NLRs protein is featured with a nucleotide-binding site (NBS) of tripartite domain structure, which contains leucine-rich repeat genes (LRR). (Ting et al., 2008; Wilmanski et al., 2008). The C-terminal of NLRs is generally considered to be the recognition and binding domain of microbial sources ligands, which corresponds to a danger signal sensor. While the N-terminal is a variable effect domain that composed of caspase recruitment domain (CARD), a pyrin domain (PYD) and baculovirus inhibitor repeat domain (BIRD) (Bertin and DiStefano, 2000; Koonin and Aravind, 2000; Inohara and Nuñez, 2001; Martinon et al., 2001; Pawłowski et al., 2001; Staub et al., 2001). Similarly, they all have a central nucleotide-binding oligomerization domain (NOD), which could eventually lead to the oligomerization and activation of NLRs (Birnbaum et al., 1994; Fairbrother et al., 2001; Weber and Vincenz, 2001; Tschopp et al., 2003). Synchronously, NLRs also could be subdivided into several subfamilies depending on the difference of N-terminal effector domains, such as CARDs. CARD domain containing 5 (NLRC5) is one of the largest members of the CARDs family (Ting et al., 2008). Petrus et al. initially revealed the precise structural feature of the caspase recruitment domain of mouse NLRC5 and subsequently indicated that atypical CARD of mouse NLRC5 and the tandem CARD mouse RIG-I might interact through the hydrophobic surface formed by α-helices 1 and 6 and the α5-α6 loop (Gutte et al., 2014). These promising discoveries may contribute to designing and synthesizing novel small molecular compounds targeting NLRC5. For another, microbial components, NLRC5 expression, and the pro-inflammatory cytokines have been respectively detected in a variety of immune tissues and cells of inflammatory disease. Simultaneously, these studies also indicated that NLRC5 may play a crucial role in specifically recognizing pathogenic microorganisms of non-self and negatively regulating the expression of inflammatory cytokines (Benko et al., 2010; Cui et al., 2010; Kuenzel et al., 2010; Meissner et al., 2010). Besides, the gene of NLRC5 has been found in many species, such as humans, mice, horses, and pig (Meissner et al., 2012). On one hand, some exploratory research has revealed that NLRC5 mRNA expression decreased slightly in immune diseases. (Kuenzel et al., 2010). On the other hand, several studies have also shown that the mRNA expression of NLRC5 increased dramatically in human and mouse immune tissues of immune disease, including bone marrow, lymph nodes, thymus, and spleen (Neerincx et al., 2010; Davis et al., 2011). To sum up, NLRC5 predominantly exists in bone marrow, human THP-1 cells, B cells, human cervical cancer cell line, and primary cells of myeloid and lymphoid origin. Based on advanced research, we made an in-depth discussion about the expression of NLRC5 in immune tissues and cells of different species, which influenced by inflammation (**Table 2**). The gene of NLRC5 locates in human chromosome 16q13, which straddles about 94 kbp and encodes a protein with molecular mass of 204 kDa. Although it is not absolute, the NLRs are mainly localized to the cytoplasm and then play a vital role in recognition of pathogen and host defense (Franchi et al., 2009). The previous studies have also suggested that NLRC5 is predominantly localized in the cytoplasm rather than the nucleus or mitochondria in 293T cells transfected with NLRC5-GFP fusion DNA (Cui et al., 2010). Furthermore, some studies have indicated that NLRC5 has similar properties of transcriptional regulation with CIITA, which also may be a nuclear localization protein molecule that could shuttle between the nucleus and the cytoplasm. In fact, it was observed that NLRC5 was located in the nucleus in normal physiological condition. Also, NLRC5 with high expression tends to be localized in the nucleus when Leptomysin B, an inhibitor of CrmA-dependent nuclear export, was used to stimulate cells (Benko et al., 2010; Meissner et al., 2010). Generally speaking, NLRC5 is mainly located in the cytoplasm when its expression is promoted. On the contrary, NLRC5 with physiological expression is often located in the nucleus.

**Table 2:** The expression of NLRC5 in various species influenced by inflammation.

Species	Tissues/Cells	Upregulated/Downregulated	Pathways	Author, Year
Chicken	Macrophages	Upregulated	IFNA and IFNB	Lian et al., 2012
Mice	Peritoneal macrophages	Upregulated	NF-κB/IFN-1	Tong et al., 2012
Mice	RAW264.7	Upregulated	JAK2/STAT3	Li et al., 2014
Chicken	Spleen	Upregulated	NF-κB	Chang et al., 2015
Human	LX-2 cells	Upregulated	NF-κB/Smad3	Xu et al., 2015
Human	HCC tissues and cells	Upregulated	AKT/VEGF-A	He et al., 2016
Human	Cervical cells	Upregulated	MiR/NF-κB	Li et al., 2016a; Li et al., 2016b
Mice	HSC cells	Upregulated	NF-κB	Liu et al., 2016
Human	Keloid fibroblasts	Upregulated	TGF-β1/Smad	Ma et al., 2016
Human	HCC tissues and cells	Upregulated	Wnt/β-catenin	Peng et al., 2016
Fish	Atlantic Salmon	Upregulated or Downregulated	NLRC5 inflammasome	Pontigo et al., 2016
Chicken	Embryo fibroblasts	Downregulated	MHC-I	Qiu et al., 2016
Mice	Liver tissues and HSC	Upregulated	NF-κB	Xu et al., 2016
Rat	FLSs	Upregulated	NF-κB	Liu et al., 2017b
Fish	Zebrafish	Upregulated	IFN-1	Wu et al., 2017
Fish	Rainbow trout	Upregulated or Downregulated	unknown	Alvarez et al., 2017
Mice	Cardiac muscle cells	Downregulated	TLR4/NF-κB	Ma and Xie, 2017
Rat	Cardiac fibroblasts	Upregulated	TGF-β1/Smad3	Zhou et al., 2017
Human	HK-2	Upregulated	PI3K/AKT	Han et al., 2018
Mice	Hepatocyte	Upregulated	Noncoding RNA	Wang et al.,2018
Human	Renal biopsy samples	Upregulated	TGF-β1/Smad	Wang et al., 2018
Human	Blood samples	Downregulated	Methylation	Zeng et al., 2018

## Biological Functions

### NLRC5’s Role in Immune Responses

#### Antiviral Immune Response

There is growing evidence that members of the NLRs family play a key role in antiviral responses. In fact, NLRC5 was demonstrated to be the last prosecutor of NLRs in the presence of virus invasion (Lupfer and Kanneganti, 2013). It was also reported that NLRC5 was involved in the modulation of innate antiviral immune response. For instance, it has been demonstrated that NLRC5 effectively prevents viral infection by inhibiting the activation of RIG-I and MDA5 as well as the production of type I IFN (Lupfer and Kanneganti, 2013; Mehraj et al., 2013). The expression of NLRC5 was increased in the STAT1 dependent manner after being stimulated by IFN-b or IFN-c (Wu et al., 2017). Nonetheless, many cell lines and primary human fibroblasts that have knocked out the NLRC5 gene exhibited a significant decrease of IFN-a/b levels after virus stimulation. This finding suggested that NLRC5 may obviously enhance antiviral signaling (Ranjan et al., 2015). For another, Kumar H et al. found that cytokines production of macrophages and DCs are not affect by virus, HSV-1, or polyI:C infection (Kumar et al., 2011). At present, the antiviral immune response of NLRC5 is still unclear, and further studies are needed to reveal the potential mechanism.

#### Inflammatory Immune Response

NLRs could recognize intracellular pathogens and promote the expression of pro-inflammatory cytokines. A great number of studies have suggested that NLRC5 is widely expressed in immune tissues, lymphocytes, and macrophages/monocytes (Benko et al., 2010; Lian et al., 2012). Furthermore, NLRC5 could play an important role in the regulation of inflammatory pathways (Ciraci et al., 2010; Davis et al., 2011; Pacifico and Davis, 2016). For example, NLRC5 shields T lymphocytes from NK-cell-mediated elimination under inflammatory conditions (Ludigs et al., 2016). It was generally reported that NLRC5 plays an active role in the first stage of inflammation. While the importance of NLRC5 was gradually diminished with the process of inflammation (Null, 2003). Collectively, NLRC5 was an indicator that may be used as a biomarker for inflammatory disease in clinical practice.

#### NLRC5’s Genotype in Inflammatory Immune Responses

It is well known that genetic background has an effect on gene expression (Williams et al., 2007). The NLRC5 gene is located at 16q13, which is a leucine-rich repeat sequence that made up of 49 exons (94kpb) (Zupin et al., 2017). Zupin et al. also found that the rs289723, located in NLRC5 gene, was closely associated with an increased risk of chronic mild periodontitis and chronic local periodontitis. Periodontitis is a typical inflammatory disease that involves complex pathological processes (Pihlstrom et al., 2005). It was also found that the rs28972A/A genotype of NLRC5 was highly associated with susceptibility to chronic mild and chronic local periodontitis. The previous studies have shown that about 20 genes were involved in the activation of inflammation (Blander and Sander, 2012). In addition, Marth et al. found that the expression of the NLRC5 increased significantly within 3 hours in response to the introduction of E. coli. And this finding also indicated that the introduction of E. coli into the uterus of healthy animal has an effect on inducing inflammation, which was an effective model of innate immunity as well (Marth et al., 2016). Moreover, numerous studies have provided evidence that common variations in the NLR genes were highly correlated to the incidence of intestinal inflammation as well as susceptibility to cancer (Ogura et al., 2001; Villani et al., 2009; Kutikhin, 2011; Jostins et al., 2012). These findings may give us a hint that more immune diseases are closely related to NLRC5 gene phenotype and expression.

### NLRC5’s Role in Liver Diseases

#### Liver Fibrosis

NLRC5 is widely expressed in the liver tissues and has recently been recognized as an active regulator of NF-κB signaling pathways (Cui et al., 2010; Tong et al., 2012; Zhang et al., 2019). It has been reported that NF-κB could effectively modulates hepatic fibrogenesis by regulating hepatocyte injury, inflammatory signals, and fibrogenic responses (Luedde and Schwabe, 2011). Furthermore, there is increasing evidence that NLRC5 plays a key role in the development and reversal of hepatic fibrosis. Liu et al. found that the expression of NLRC5 increased during the development of liver fibrosis and decreased during the reversal stage. Besides, enforced expression of NLRC5 dramatically could inhibit the fibrosis recovery, while knockdown of NLRC5 promoted the fibrosis recovery (Liu et al., 2016). Xu et al. found that the expression of NLRC5 increased significantly in fibrotic liver tissues of human, and several components of NLR inflammasome were also found to be increased (Gressner et al., 2002). An apparently increased expression of NLRC5 in HSC was observed after being stimulated by TGF-β1, which is known as a potent stimulus for HSC-induced fibrogenesis (Xu et al., 2016). Collectively, NLRC5 may take an active part in the occurrence and reversal of hepatic fibrosis. These findings also provide essential proof of the principle that NLRC5 may represent a target for the prevention or treatment of liver fibrosis.

#### Hepatocellular Carcinoma

Pattern recognition receptors (PRRs) are innate immunity sensors that performed a pivotal role in the modulation of tumor immunity. They could identify danger signals, clean the profitless cells, and balance the quantity of the host flora to thereby modulate the tumor immunity. In addition, PPRs were also found to facilitate tumorigenesis by reducing the population of regulatory cell and inducing the immunosuppressive cytokines (Zaki et al., 2011; Pradere et al., 2014). A lot of members of NLRs have been demonstrated to participate in tumor genesis as well (Hugot et al., 2001; Da Silva Correia et al., 2006; Ohno et al., 2008; Villani et al., 2009; Zaki et al., 2011; Burdette et al., 2012). What’s more, increasing number of studies also reported that NLRC5 was involved in the development of keloid disease, human papillomavirus, human prostate cancer, and gastric cancer (Castano-Rodriguez et al., 2014; Carretero et al., 2016; Li et al., 2016a; Ma et al., 2016). Of note, the expression of NLRC5 was higher in HCC tissues than in normal tissues. Basic experiments research suggested that silencing NLRC5 could subsequently inhibit HCC cells proliferation, migration, invasion, and tumor formation. Conversely, enforced expression of NLRC5 could facilitate the proliferation, migration, and invasion of HCC cells (Peng et al., 2016). He et al. also found that NLRC5 was expressed highly in clinical samples of HCC patients. Taken together, NLRC5 may become a favorable option for treating HCC by inhibiting cancer metastasis and proliferation. (He et al., 2016).

#### Liver Inflammatory Injury

Accumulating evidence showed that persistent inflammation leads to liver injury and continuous activation of hepatic stellate cells (HSC) will make liver injury worsen. It is generally known that HSC can be activated and transformed into myofibroblast-like cells. Damage to hepatocytes and Kupffer are commonly acknowledged as the first-line effects during the activation of quiescent HSC (Allen, 2011; Mormone et al., 2011). It was also demonstrated that the level of NLRC5 expression could act as an indicator of inflammation severity. TNF-α, a super innate immune activator, could significantly increase the expression of NLRC5 in HSC (Fan et al., 2013; Koppula et al., 2013). Besides, Li et al. found that NLRC5 expression could be quickly induced by LPS. Also, they hypothesized that NLRC5 may play a pivotal role in LPS-induced cytokine secretion of hepatic macrophages (Li et al., 2014). Furthermore, Xu et al. also found that NLRC5 is responsible for inflammatory cytokine expression by promote the secretion of inflammatory factors in LX-2 cells (Xu et al., 2015). In conclusion, these findings suggested that the regulation of NLRC5 expression may be of great significance in the treatment of inflammatory liver injury.

#### Hepatic Steatosis

A large number of studies have shown that hepatic steatosis is a key event in the process of liver injury induced by EtOH. Alcoholic steatosis is one of the major causes of alcoholic liver disease (Bieghs et al., 2013). Alcoholic liver disease (ALD) is a worldwide common disease with high morbidity and mortality, which is closely associated with excessive alcohol consumption (Ricieri Brito et al., 2010; Dubuquoy, 2016; Zhang et al., 2018). Functional studies have suggested that NLRC5 could positively regulate ethanol-induced hepatic steatosis. Wang et al. reported that the expression of NLRC5 increased significantly in EtOH-fed mice, and it also may be involved in the pathogenesis of EtOH-induced hepatic steatosis (Wang et al., 2018b).

#### Liver Ischemia/Reperfusion Injury

**Figure 1:**
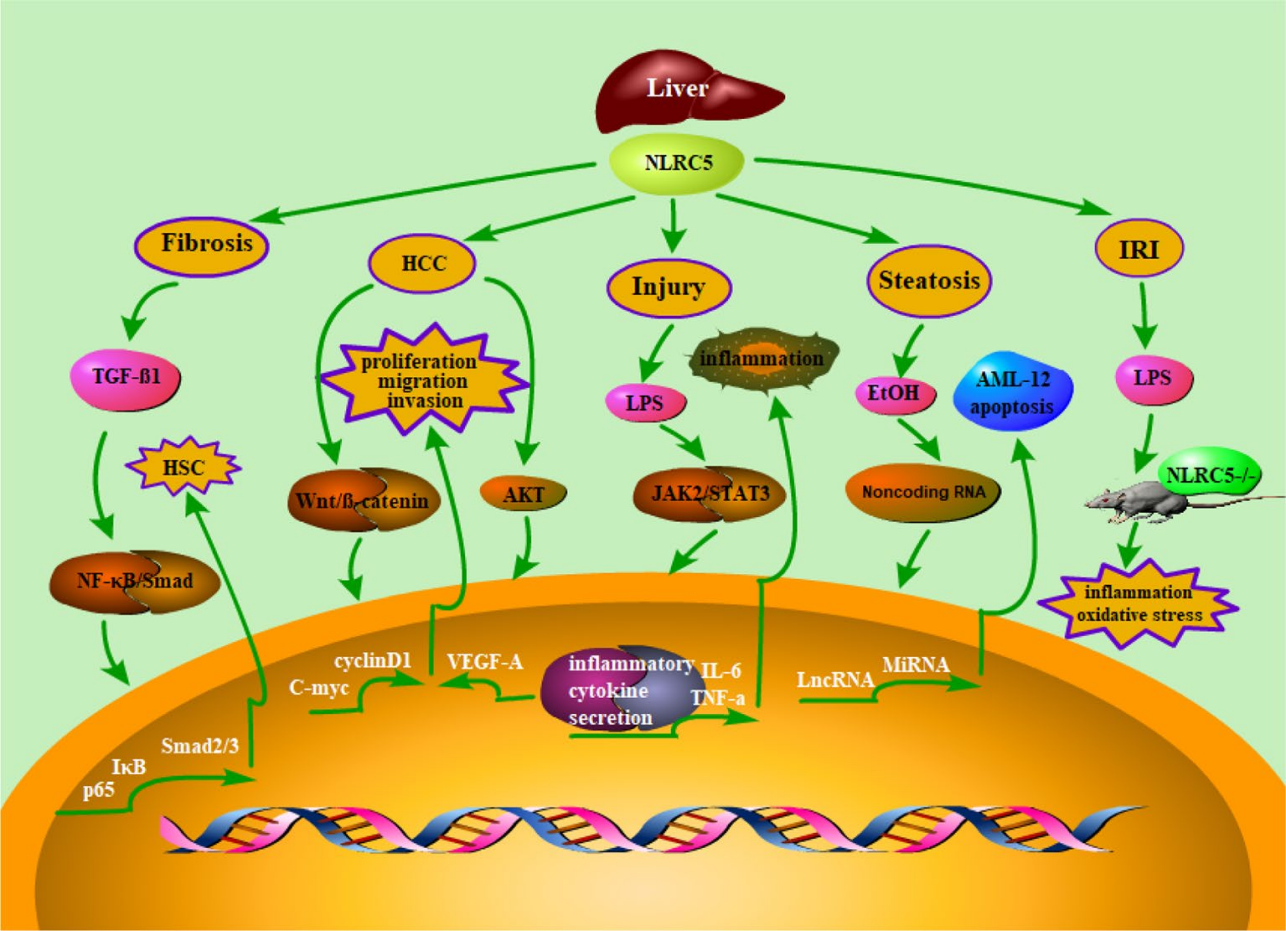
Schematic representation of NLRC5’s role in liver diseases. Accumulating evidence suggested that NLRC5 has been linked to a lot of liver diseases, such as liver fibrosis, hepatocellular carcinoma, liver inflammatory injury, hepatic steatosis, and Liver ischemia/reperfusion injury. Moreover, it has been demonstrated that NLRC5 plays an essential role in regulating relevant mechanisms of liver diseases. Herein, we summarized the NLRC5-relevant signaling pathways, which were involved in liver diseases.

Ischemia reperfusion injury (IRI) of liver is an unavoidable clinical event during liver surgery, such as hepatectomy, hypovolemic shock, and liver transplantation (Aldrighetti et al., 2006; Linares et al., 2018; Zhong et al., 2018). A large number of inflammatory cytokines were released in the process of liver ischemia/reperfusion injury, and then they can quickly lead to hepatocyte damage (Van Golen et al., 2013). Given that NLRC5 plays an important role in the regulation of inflammation, it may also be involved in the regulation of liver ischemia/reperfusion injury. Chen et al. reported that NLRC5-deficient mice developed severe liver damage and inflammatory response after I/R surgery (Chen et al., 2017). In short, these findings suggested that NLRC5 may play a vital role in alleviating liver I/R injury (**Figure 1**).

### NLRC5’s Role in Renal Diseases

#### Renal Fibrosis

Renal fibrosis is considered to be a representative pathological character in the development of chronic renal disease, which is a worldwide public health event with high mortality (Levey et al., 2007; Liu, 2011). It is characterized by the accumulation and deposition of extracellular matrix (ECM) proteins, which mainly caused by the increase of myofibroblasts (Strutz and Zeisberg, 2006). One study has shown that NLRC5 could act as a fibrogenic molecule, which involves in the occurrence and development of fibrosis diseases (Xu et al., 2016). Besides, Wang et al. found that the expression of NLRC5 increased significantly in renal fibrosis tissues and cells. Knockout of NLRC5 dramatically could inhibit the proliferation of renal fibrosis cells and decrease the accumulation of ECM (Wang et al., 2018c). These findings strongly support the view that NLRC5 participate in the pathogenesis of renal fibrosis and can also be treated as a therapeutic target.

#### Acute Kidney Injury

Acute kidney injury is a common clinical complication, which is mainly caused by renal ischemia-reperfusion (I/R) injury (Fanelli et al., 2018; Lazzeri et al., 2018; Wang et al., 2018a). Tubular epithelial cell injury is the most prominent feature of acute renal injury (Liu et al., 2015). Exposure of human renal tubular epithelial cells (HK-2) to H/R is a classic model of renal I/R injury model for study *in vitro*. Han et al. found that NLRC5 is highly expressed in HK-2 cell of H/R injury model. It has also been shown that silencing NLRC5 obviously increased the viability of H/R injury HK-2 cell, inhibited the apoptosis of renal I/R injury cell and promoted the recovery of renal I/R injury (Han et al., 2018). Quanxin Li and colleagues identified that serum creatinine and renal tubule injury were significantly reduced in the renal I/R injury of NLRC5 defective mice. Their results strongly support the hypothesis that NLRC5 plays a leading role in renal I/R injury. Therefore, the regulation of NLRC5-mediated pathway may be of great significance in the treatment of acute renal injury (Li et al., 2018b). In addition, these studies also indicated that acute kidney injury can be reversed by down-regulating the expression of NLRC5.

#### Diabetic Nephropathy

**Figure 2:**
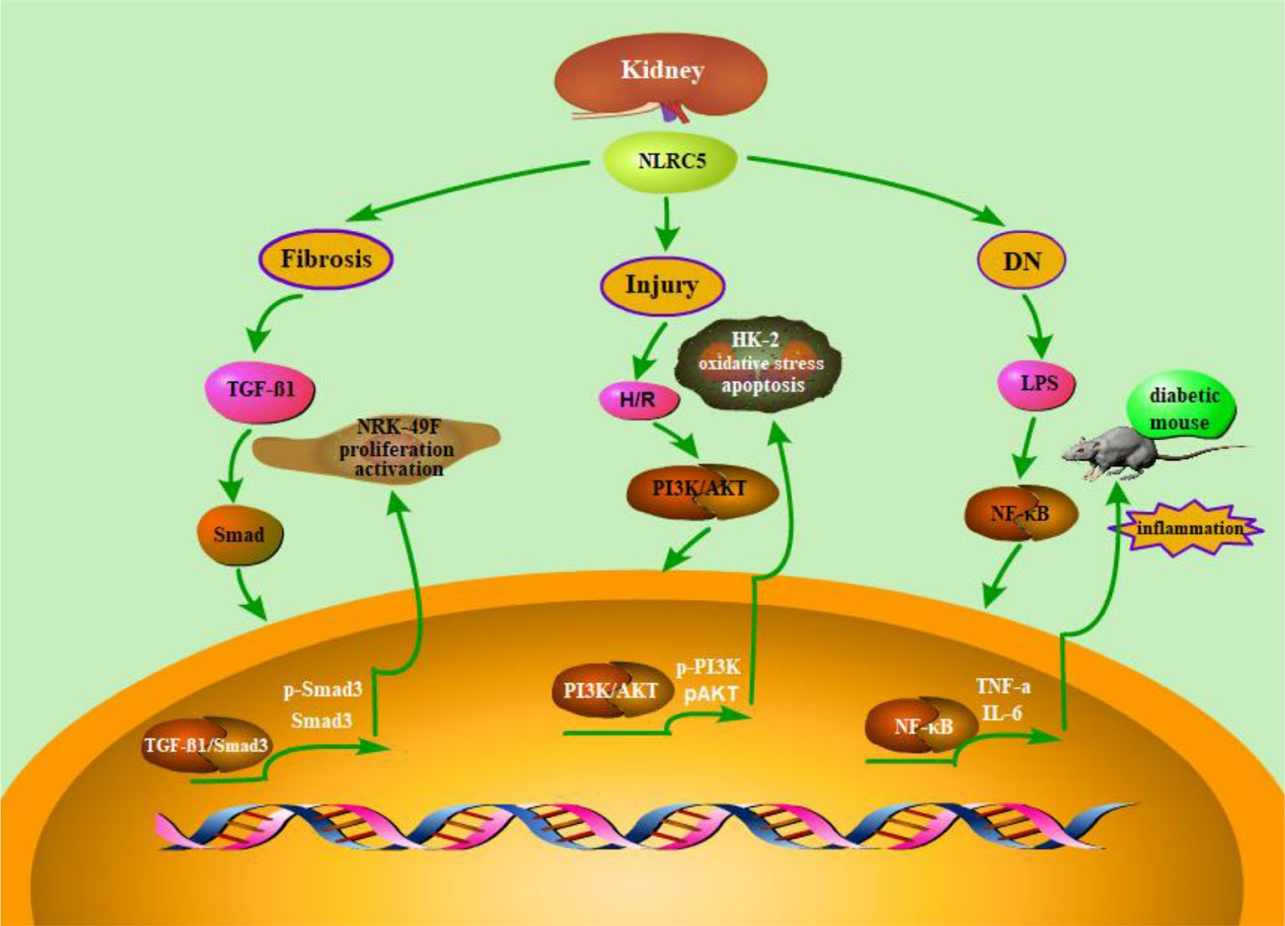
Schematic representation of NLRC5’s role in renal diseases. As is described in the above content, NLRC5 is widely expressed in renal related tissue and has also been demonstrated to involve in a variety of kidney diseases for and wide. A number of functional studies have reported that Up-regulation or down-regulation of NLRC5 may play a key role in the underlying pathogenesis of renal disease. For instance, knock out of NLRC5 could inhibit the proliferation of renal fibrosis cells and decrease the accumulation of ECM. Besides, RNA interference-mediated Knock down of NLRC5 attenuates renal I/R injury *in vitro* through the activation of PI3K/Akt signaling pathway.

Diabetic nephropathy (DN) is one of the major causes of advanced nephropathy and has become a worldwide public health problem (Saran et al., 2017; Li et al., 2018a; Mathur et al., 2018). Pathological characteristics of DN include oxidative stress, inflammatory responses, and metabolic dysfunction, while the exact pathogenesis are complex and still remain to be further investigated (Wada and Makino, 2016). Recent studies have reported that innate immunity plays an essential role in the pathogenesis of DN (Navarro-Gonzalez et al., 2011). In addition, NLRC5 is demonstrated to participate in the occurrence and development of DN (Luan et al., 2018). It has been demonstrated that NLRC5 is highly expressed in DN. And knocking out of NLRC5 could promote the recovery of kidney injury in diabetic mice. These studies suggested that NLRC5 plays an important role in inflammatory responses and fibrosis development, which could make diabetic kidney deteriorate. These findings also emphasize the importance of NLRC5 in the progression of diabetic nephropathy. Therefore, down-regulating the expression of NLRC5 is a potential approach for DN treatment by alleviating inflammatory response (**Figure 2**).

### NLRC5’s Role in Rheumatoid Arthritis

Rheumatoid arthritis (RA) is a chronic autoimmune disease, which could eventually cause joint deformities and disability (Rossato et al., 2015; Zhang et al., 2015; Zhang et al., 2016a). Fibroblast-like synoviocytes (FLSs) are at the junction of the joint and the abnormal proliferation of FLSs is a central factor in the progression of RA (Jia et al., 2016; Li et al., 2016b). IL-17 is commonly considered to be a key proinflammatory cytokine in RA pathogenesis (Buckland, 2010). It has also been reported that IL-17 participated in the pathogenesis of inflammatory diseases through the NLRP3-mediated inflammasome pathways (Cho et al., 2012; Chi et al., 2017). Besides, previous studies have shown that NLRs are wildly expressed in RA, and they also play an important role in inflammatory response (Theofilopoulos et al., 2010; Takakubo and Konttinen, 2012). Since NLRs involve in inflammatory diseases and promote the clearance of invasive pathogens, it may also be involved in the progress of RA. It is well known that Animal model of Adjuvant arthritis (AA) is a typical model for studying RA (Liu et al., 2017a; Sun et al., 2017). Liu et al. found that NLRC5 is highly expressed in AA rat animal. Moreover, RNA interference-mediated Knock down of NLRC5 could significantly inhibit the proliferation of FLSs and reduce the expression of inflammatory cytokine (Liu et al., 2017b). And thus, NLRC5 may be a potential therapeutic target for the treatment of RA. For instance, we can effectively inhibit the RA progression by regulating the expression of NLRC5.

### NLRC5’s Role in Heart Diseases

Cardiac fibrosis is one of the most obvious pathological characteristics of myocardial remodeling in heart diseases, which has a high morbidity and mortality (Krenning et al., 2010; Burlew and Weber, 2014). Because of its complexity and multifactor, the molecular mechanism of myocardial fibrosis remains to be further illuminated (Brilla et al., 2000; Spinale et al., 2000; Leask, 2010). The major pathological features of cardiac fibrosis are rapid proliferation of CFs and excessive deposition of extracellular matrix (ECM) (Porter and Turner, 2009). Zhou et al. found that NLRC5 was highly expressed in TGF-βl-induced CFs (Zhou et al., 2017). Recent advance have also found that silencing NLRC5 could significantly inhibit proliferation, ECM deposition, and pro-fibrotic molecules expression in CFs (Yang et al., 2019). These results initially suggested that NLRC5 has a regulatory effect on accelerating myocardial fibrosis, which involves in heart disease. Therefore, down regulating the expression of NLRC5 may be useful to alleviate myocardial fibrosis. For another, Ma et al. reported that a high-fat diet can lead to myocardial injury, which was particularly evident in mice with the NLRC5 deficiency (Ma and Xie, 2017). They also found that the deficiency of NLRC5 increased the expression of fibrosis-related proteins. At the same time, the study of cardiac function markers also indicated that NLRC5 knock out obviously induced heart dysfunction. In conclusion, these evidences indicated that the level of NLRC5 expression is closely related to heart disease. Indeed, further exploratory and validation research are urgently needed to illustrate the substantial link between NLRC5 and heart disease.

### NLRC5’s Role in Lung and Spleen Diseases

The gene of human NLRC5 is highly expressed in lung and spleen (Biswas et al., 2012; Yao and Qian, 2013). NLRC5 is famous as a transactivator of MHC class I and also plays a central role in tumor immune escape through regulation of MHC class I (Garcia-Lora et al., 2003; Staehli et al., 2012; Li et al., 2019). Li et al. investigated the role of NLRC5 in patients with stage III non-small-cell lung cancer (NSCLC) (Li et al., 2015). They found that NLRC5 is highly expressed in NSCLC tissues. Since decreased nuclear expression of NLRC5 is related to the loss of MHC class I heavy chain, NLRC5 also be considered as a prognostic indicator and a predictor of survival in NSCLC patients (Bijen et al., 2010). And thus, the level of NLRC5 expression could be used to calculating the survival of NSCLC patients. Besides, Guo et al. found that upregulation of NLRC5 induced airway epithelial cell proliferation. Triantafilou et al. also found that virus infection in primary bronchial cells significantly induced the expression of NLRC5 (Triantafilou et al., 2013; Guo et al., 2015). Chang et al. reported that the mutation of NLRC5 promoter was involved in the modulation of related signaling pathways after chicken spleen infection (Chang et al., 2015). Indeed, our understanding of NLRC5′ role in lung and spleen diseases is extremely poor and remains to be improved.

## Regulation Mechanism

### The Methylation of NLRC5

It has been reported that DNA methylation plays an important role in genomic dynamics, which is closely related with development of various diseases. It may change at any one site, and thus affect many biological processes of human disease (Bernstein et al., 2007; Feinberg, 2007; Lister et al., 2009). An accurate and in-depth understanding of the DNA methylation status of genes is essential to uncover the underlying DNA methylation mechanism and pathogenic mechanism (Li et al., 2010). Besides, it is also known that methylation of NLRC5 is negatively correlated with MHC class I gene expression. Furthermore, the expression of NLRC5 is demonstrated to be regulated by NLRC5 methylation (Guo et al., 2016). Zeng et al. investigated the NLRC5 methylation sites and status in the genome of centenarians and subsequently indicated that hypomethylation of NLRC5 are highly correlated with age in centenarians (Zeng et al., 2018). Previous studies found that the degree of NLRC5 methylation is negatively correlated with NLRC expression in a variety of cells (Yoshihama et al., 2016; Yoshihama et al., 2017). Zhang et al. found that HIV infection can lead to lower methylation of NLRC5, suggesting that NLRC5 plays an important role in the pathogenesis of HIV (Zhang et al., 2016b). Taken together, these evidences indicated that NLRC5 methylation plays a key role in immune diseases by regulating multiple biological pathways, and it could act as a promising biomarker as well.

### NLRC5 Inflammasome

The formation of NLRC5 inflammasome is of great importance to recognize pathogens, activate inflammatory caspases, and modulate host defence response (Martinon et al., 2009). Davis et al. thought that NLRC5 could form inflammasome in combination with NLRP3 in the presence of pathogens (Davis et al., 2011). It has been revealed that RNA interference-mediated knockdown of NLRC5 significantly decrease caspase 1, IL-1β, and IL-18 expression, which are response to pathogens infection. Therefore, they hold the view that NLRC5 is an indispensable component in the secretion of inflammasome dependent inflammatory cytokine. It has also been suggested that NLRC5 cooperates with NLRP3 to reconstitute inflammasome activity in an ectopic system (Ting and Davis, 2005). Besides, NLRC5 is widely expressed in tissues with bacterial and viral infections, and it can also induce expression of inflammasome-related proteins (Livak and Schmittgen, 2001). It was also reported that NLRC5 inflammasome involves in parr and smolt stage of Atlantic salmon. To sum up, these studies suggested that NLRC5 may be involved in the regulation of immune disease by the way of forming a functional inflammasome.

### Signaling Pathways

**Figure 3:**
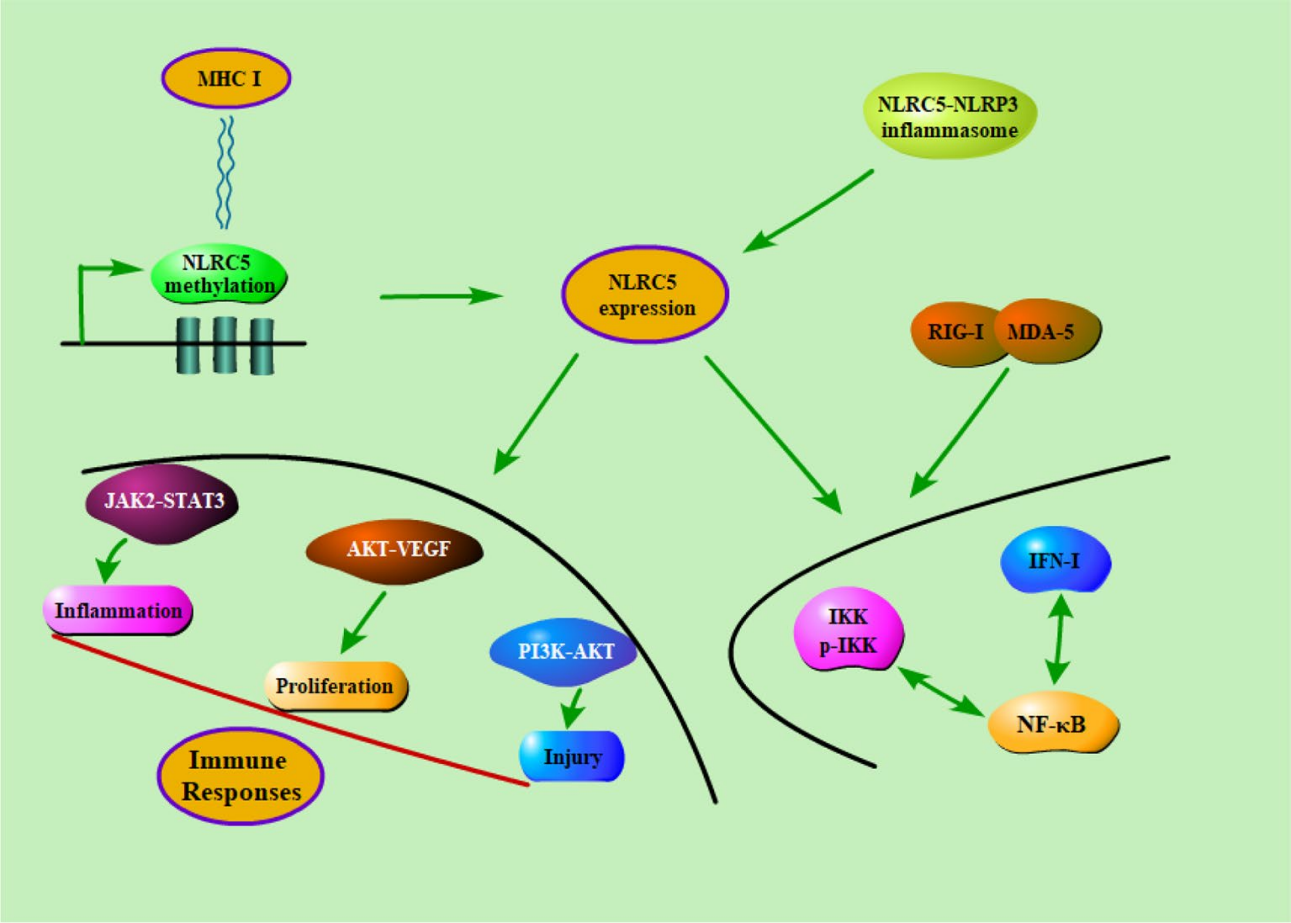
The potential regulatory mechanisms of NLRC5 in immune diseases. Firstly, the methylation of NLRC5 were negatively correlated with MHC class I gene expression. Secondly, NLRC5 could form inflammasome in combination with NLRP3 in the presence of pathogens. Finally, NLRC5 could modulate immune response through a series of signaling pathways that have already been confirmed to participate in the occurrence and development of immune diseases for and wide.

NF-κB is considered to be one of the most important transcription factors associated with inflammatory diseases. It is known that NF-κB and type I interferon signaling pathways could be directly regulated by themselves through the modulation of feedback loops (Komuro et al., 2008). NLRC5 plays a regulatory role in inflammatory response, innate immune response, and antiviral response. It was reported that NLRC5 could combine with RIG-I and MDA5 to encompass IFN-I pathway. In short, NLRC5 has been demonstrated to play a pivotal role in activating type I interferon signaling pathways. Of course, in one way, IFN-γ could regulate the promoter of NLRC5 and its gene product. For another, NLRC5 is considered as a mediator that could positively or negatively regulate IFN, NF-κB signaling pathways. (Cui et al., 2010). They found that NLRC5 inhibited the phosphorylation of IKK and frustrated the activation of NF-κB. Furthermore, some studies also suggested that RNA interference-mediated knockdown of NLRC5 significantly enhanced the signaling of NF-κB and type I IFN in mice. On the contrary, multiple studies implicated that NLRC5 positively regulated NF-κB and IFN-I pathways. For instance, knockdown of NLRC5 lead to reduced signaling of NF-κB and type I IFN, and up-regulating the expression of NLRC5 promoted IKK and IRF3 phosphorylation followed by activation of NF-κB (Neerincx et al., 2012; Tong et al., 2012). In addition, NLRC5 could modulate immune response through many signaling pathways that have already been confirmed to participate in the occurrence and development of immune diseases for and wide. Meissner et al. found that NLRC5 significantly increased the expression of MHC class I by activating the promoters of MHC class I genes (Meissner et al., 2010). Li et al. also found that NLRC5 induced the secretion of the inflammatory cytokine through JAK2/STAT3 signaling pathway (Li et al., 2014). It has been reported that NLRC5 promoted the cell proliferation, which is likely to be mediated by the AKT/VEGF-A pathway (He et al., 2016). Han et al. also demonstrated that NLRC5 could accelerate renal I/R injury by activating PI3K/AKT signaling pathway (Han et al., 2018). In short, these findings indicated that NLRC5 was involved in the occurrence and development of immune disease through regulation of diverse signaling pathways (**Figure 3**).

## Concluding Remarks

As reviewed above, the diverse characters of NLRC5 have been introduced and summarized. In addition, recent advance about immanent characteristics, biological function of NLRC5 and molecular mechanisms of NLRC5-mediated immune diseases are also discussed. In the past decades of years, researchers have made much progress in characterizing the NLRC5 crystal structure and detecting the expression of NLRC5 in tissues and cells of immune disease. The fact that NLRC5 plays an essential role in the control of the regulatory mechanism has made it a promising therapeutic target for immune disease. The role of NLRC5, however, still remains controversial in innate immunity and relevant signaling pathways. In a word, the relevant pathways of NLRC5 in innate immunity have not yet been elucidated clearly so far and more experimental investigations about how NLRC5 performs in the host defense against pathogens and transmits immune signals are urgently needed in the future. Moreover, there are few studies on the role of NLRC5 in human immune diseases. Therefore, it is necessary to put on a deep insight on the relationship between the immune function of NLRC5 and human immune diseases. Besides, given that the swift development of molecular docking technology, more precise characterization of crystal deserved to be explored specifically. In summary, these promising properties of NLRC5 make it an appealing therapeutic target through regulating the expression of NLRC5 and NLRC5-dependent signaling pathways.

## Author Contributions

J-QW, Y-RL, and JLi wrote the main manuscript text. QX and JLia prepared **Figures 1**–**3**. Q-RX and R-NC prepared **Tables 1** and **2**. All authors reviewed the manuscript.

## Conflict of Interest

The authors declare that the research was conducted in the absence of any commercial or financial relationships that could be construed as a potential conflict of interest.
